# Survival of patients with metastatic leiomyosarcoma: the MD Anderson Clinical Center for targeted therapy experience

**DOI:** 10.1002/cam4.956

**Published:** 2016-11-23

**Authors:** Zhijie Wang, Naiyi Shi, Aung Naing, Filip Janku, Vivek Subbiah, Dejka M. Araujo, Shreyaskumar R. Patel, Joseph A. Ludwig, Lois M. Ramondetta, Charles F. Levenback, Pedro T. Ramirez, Sarina A. Piha‐Paul, David Hong, Daniel D. Karp, Apostolia M. Tsimberidou, Funda Meric‐Bernstam, Siqing Fu

**Affiliations:** ^1^ Department of Investigational Cancer TherapeuticsThe University of Texas MD Anderson Cancer CenterHoustonTexas; ^2^ Department of Medical OncologyCancer HospitalChinese Academy of Medical Sciences & Peking Union Medical CollegeBeijingChina; ^3^ Department of Sarcoma Medical OncologyThe University of Texas MD Anderson Cancer CenterHoustonTexas; ^4^ Department of Gynecologic Oncology and Reproductive MedicineThe University of Texas MD Anderson Cancer CenterHoustonTexas

**Keywords:** gene aberration‐related therapy, leiomyosarcoma, overall survival, phase I clinical trial, prognostic scoring model

## Abstract

Advanced stage leiomyosarcoma (LMS) is incurable with current systemic antitumor therapies. Therefore, there is clinical interest in exploring novel therapeutic regimens to treat LMS. We reviewed the medical records of 75 consecutive patients with histologically confirmed metastatic LMS, who had been referred to the Clinical Center for Targeted Therapy at MD Anderson Cancer Center. To lay the foundation for potential phase I trials for the treatment of advanced LMS, we analyzed tumor response and survival outcome data. The frequent hotspot gene aberrations that we observed were the *TP53* mutation (65%) and *RB1* loss/mutation (45%) detected by Sequenom or next‐generation sequencing. Among patients treated with gene aberration‐related phase I trial therapy, the median progression‐free survival was 5.8 months and the median overall survival was 15.9 months, significantly better than in patients without therapy (1.9 months, *P *=* *0.001; and 8.7 months, *P *=* *0.013, respectively). Independent risk factors that predicted shorter overall survival included hemoglobin <10 g/dL, body mass index <30 kg/m^2^, serum albumin <3.5 g/dL, and neutrophil above upper limit of normal. The median survivals were 19.9, 7.6, and 0.9 months for patients with 0, 1 or 2, and ≥3 of the above risk factors, respectively (*P *<* *0.001). A prognostic scoring system that included four independent risk factors might predict survival in patients with metastatic LMS who were treated in a phase I trial. Gene aberration‐related therapies led to significantly better clinical benefits, supporting that further exploration with novel mechanism‐driven therapeutic regimens is warranted.

## Introduction

Leiomyosarcoma (LMS) is a malignant mesenchymal tumor originating from smooth muscle tissues 1. LMS is relatively rare representing 10–20% of soft tissue sarcomas 2. It can appear at almost all anatomic sites such as the uterus 3, retroperitoneum 4, extremities 5, and blood vessels 6. Uterine LMS is the most common site‐specific group with an estimated incidence of 0.64 cases per 100,000 women 7. Pathologically, LMS possesses a typical histologic pattern of intersecting and sharply marginated fascicles of spindle cells with abundant eosinophilic cytoplasm and elongated and hyperchromatic nuclei 1, 2, 8.

Surgical resection of localized disease is a well‐established therapeutic strategy 9. In the event tumors have metastasized, hormonal therapy 10 and cytotoxic chemotherapeutic agents such as gemcitabine 11, docetaxel 12, 13, anthracyclines 14, 15, ifosfamide 16, temozolomide 17, trabectedin 18, 19, eribulin 20, 21, and many other cytotoxic agents provide modest antitumor activity 22. In contrast, novel targeted therapeutic agents have not widely used for the treatment of advanced LMS. In a randomized phase III study (the PALETTE trial) in 372 patients with advanced nonadipocytic soft tissue sarcoma, median progression‐free survival (PFS) and overall survival (OS) were 4.6 months and 12.5 months with pazopanib compared with 1.6 months and 10.7 months, respectively, with placebo 23, which has established pazopanib as a new treatment option for patients with metastatic nonadipocytic soft tissue sarcomas including LMS after previous chemotherapy. In a randomized phase II study of patients with advanced soft tissue sarcoma, treatment with doxorubicin plus olaratumab, a monoclonal antibody against the platelet‐derived growth factor receptor alpha, led to significantly greater median OS than doxorubicin plus placebo (26.5 months vs. 14.7 months, *P *<* *0.001), associated with favorable objective responses and median PFS 24.

The aims of this study were to analyze patient demographics, molecular characteristics, and clinical outcomes in patients with metastatic LMS who had been referred to the Clinical Center for Targeted Therapy at The University of Texas MD Anderson Cancer Center, and to explore potential therapeutic regimens to treat advanced LMS, and risk factors to predict survival in these patients.

## Materials and Methods

### Patients

We retrospectively reviewed the medical records of 75 consecutive patients with histologically confirmed metastatic LMS who had been referred to the Clinical Center for Targeted Therapy at MD Anderson between 1 July 2005 and 30 September 2013. Of these, 54 patients had received phase I trial therapy. We reviewed patients’ demographics, medical history, clinical characteristics, laboratory results, gene aberrations, and status of phase I trial therapy. Trial conduct, data collection, and the subsequent analysis were performed in accordance with the guidelines of the MD Anderson Institutional Review Board.

### Molecular analysis

When available, efforts were made to provide adequate tissues for molecular analyses. Tumor DNA was extracted from microdissected paraffin‐embedded tumor specimens for gene aberration detections in a Clinical Laboratory Improvement Amendments (CLIA)‐certified Molecular Diagnostics Laboratory 25, 26. Genomic analysis for hotspot mutations was detected by Sequenom (an 11‐gene panel) or next‐generation sequencing (a 46‐gene, 50‐gene, or Foundation Medicine panel).

### Treatment and evaluation

The decision of whether to allocate an eligible patient into a phase I trial depended on protocol availability and the preference of the treating physician. Tumor responses (CR = complete remission, PR = partial response, SD = stable disease, and PD = progressive disease) were evaluated according to the Response Evaluation Criteria in Solid Tumors (RECIST version 1.0 or 1.1) 27, 28, depending on individual protocols. PFS was calculated from initiation of phase I trial therapy to the first objective documentation of PD, the time of death, or the last date censored on 10 December 2015. OS was estimated from the date of the initial visit to the Clinical Center for Targeted Therapy to death or the last date censored on 10 December 2015.

### Statistical analysis

Patient characteristics, including age, race, site of origin, Eastern Cooperative Oncology Group (ECOG) performance status score, lactate dehydrogenase (LDH), albumin, the number of metastatic sites, neutrophils, lymphocytes, hemoglobin, platelets, serum creatinine, total bilirubin, prior antiangiogenic therapy, prior systemic lines, number of protocols enrolled, and gene aberrations were summarized with use of frequency distributions and percentages. Gene aberration‐related therapy was defined when at least one drug in the regimen was known to inhibit the functional activity of at least one of the patient's gene aberration and/or its key downstream components, that is, an antiangiogenic therapy for *TP53* mutation, or an mTOR inhibitor‐based therapy for *PIK3CA* mutation. Categorical variables were compared via chi‐square and Fisher's exact tests. Survivals (PFS and OS) were assessed by using the Kaplan–Meier curve by the log‐rank test. The multivariable Cox proportional hazards model was used for multivariate analysis. All tests were two sided and considered significant when *P* values were less than 0.05. Statistical analyses were performed by using SPSS version 23.0 software (SPSS, Chicago, IL).

## Results

### Patient characteristics and molecular aberrations

In 54 patients who received phase I trial therapy, the median age was 55 years. The median follow‐up was 10 months (range, 1–63 months). Most of these patients were Caucasian and had a good ECOG performance status of 1 or better. In half of these patients with LMS, the disease originated in the uterus. Overexpression of estrogen receptor and/or progesterone receptor detected by immunohistochemical analysis was found in 52% of patients (11/21). Detailed patient characteristics are summarized in Table 1. In patients for whom molecular profiling had been performed, the most frequent hotspot gene aberrations were observed in *TP53* mutations (15/23, 65%), *RB1* mutation/loss (9/20, 45%), and *PTEN* mutation/loss (5/22, 23%), as seen in Table 2.

**Table 1:** Patient baseline demographics (*n* = 54)

Characteristics	*N* (%)^a^
Age (years)	
Median (range)	55 (31–76)
Race	
Caucasian	37 (69%)
African American	11 (20%)
Others	6 (11%)
Site of origin	
Uterus	27 (50%)
Retroperitoneal	13 (24%)
Others	14 (26%)
ECOG performance status	
0	12 (22%)
1	40 (74%)
2	2 (4%)
Venous thromboembolism	
Yes	14 (26%)
No	40 (74%)
Body mass index	
≥30 kg/m^2^	20 (37%)
<30 kg/m^2^	34 (63%)
Prior antiangiogenic therapy	
Yes	27 (50%)
No	27 (50%)
Prior systemic therapy	
Median (range)	3 (1–10)
One prior line	5 (10%)
Two prior lines	9 (13%)
≥3 prior lines	40 (77%)

ECOG, Eastern Cooperative Oncology Group.

a Unless otherwise specified.

**Table 2:** Molecular alterations in phase I patients with metastatic leiomyosarcoma (*n* = 54)

Gene aberration	No. of patients screened	No. of positive patients	%
TP53 mutation	23	15	65
ER/PR expression	21	11	52
RB1 mutation/loss	20	9	45
PTEN mutation/loss	22	5	23
CDKN2A/B mutation	21	3	14
STK11 mutation	21	2	10
BRCA2 mutation germline	16	1	6
PIK3CA mutation	33	2	6
C‐KIT mutation	30	1	3
KRAS mutation	28	0	0
NRAS mutation	25	0	0
B‐RAF mutation	29	0	0
GNAQ mutation	21	0	0

PR, partial response.

### Antitumor activity and survival

#### Protocol therapy

The 54 study patients had received 93 phase I treatments in 60 different types of phase I protocols conducted in the Clinical Center for Targeted Therapy at MD Anderson: 26 received one line only, 15 received two lines, and 13 received three lines. The 60 types of phase I protocols were classified as follows: targeted therapy as a single agent (*n* = 17), as a combination of two target agents (*n* = 17), as a single agent in combination with a chemotherapeutic agent (*n* = 13), as chemotherapy alone (*n* = 11), and as immunotherapy (*n* = 2). About 38% of protocols included an antiangiogenic agent.

#### Best tumor response

In 48 patients with measurable lesions, the initial phase I trials led to CR = 0, PR = 1, 2%, and SD = 22, 46%, which were similar to subsequent phase I therapy (*n* = 35): CR = 0, PR = 1, 3%, and SD=19, 54%. Patients treated with gene aberration‐related therapies achieved significantly higher DCR (12/13, 92%) than did those without (11/35, 31%; *P *<* *0.001). Combination therapies yielded significantly higher disease control rates (DCRs) (DCR = CR/PR/SD: 19/31, 61%) than did therapies only including a single agent (4/17, 24%; *P *=* *0.012). Antiangiogenic therapy led to CR/PR/SD (*n* = 24, 58%), compared favorably with non‐antiangiogenic therapy (*n* = 24, 33%; *P *=* *0.082).

#### Progression‐free survival

**Figure 1:**
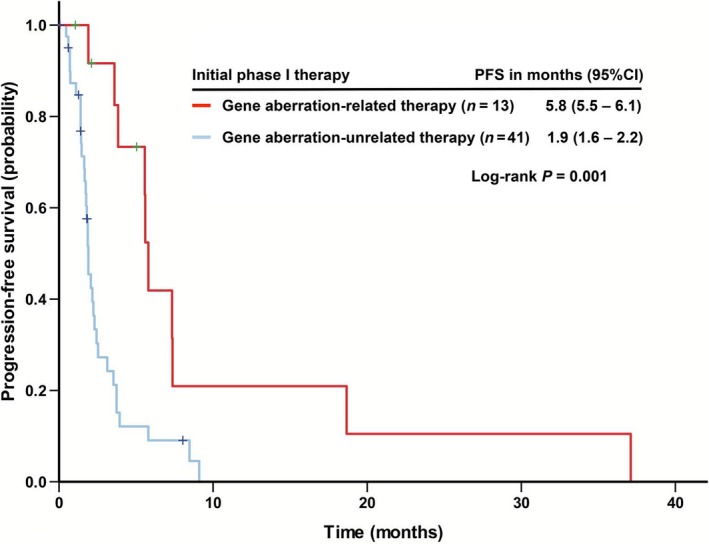
Kaplan–Meier curves of progression‐free survival (PFS) in 54 patients with metastatic leiomyosarcoma who were treated in phase I clinical trials stratified by “gene aberration‐related” or “gene aberration‐unrelated” phase I trial therapies.

The initial phase I trials led to a median PFS of 2.3 months (95% confident interval [CI], 1.6–3 months), similar to subsequent phase I trials (2.7 months [95% CI: 1.7–3.8]). Figure 1 showed that at their initial phase I trial therapy, patients treated with gene aberration‐related therapies (*n* = 13) yielded a significantly longer median PFS of 5.8 months (95% CI: 5.5–6.1) than those without (*n* = 41; 1.9 months [95% CI: 1.6–2.2]; *P *=* *0.001). Combination therapies led to a significantly longer PFS (*n* = 34, 3.5 months [95% CI: 2.1–5]) than therapies only including a single agent (*n* = 20, 1.8 months [95% CI: 1–2.6]; *P *=* *0.01). Antiangiogenic regimens yielded a median PFS of 3.7 months (95% CI: 3–4.5), significantly better than non‐antiangiogenic regimens (1.9 months [95% CI: 1.5–2.3]; *P *=* *0.03). In patients who had received antiangiogenic therapy, those with the hotspot *TP53* mutation (*n* = 10) had a median PFS of 5.8 months (95% CI: 3.3–8.3), which compared favorably with those without (*n* = 17, 2.1 months [95% CI: 0–4.2]; *P *=* *0.053).

#### Overall survival

**Figure 2:**
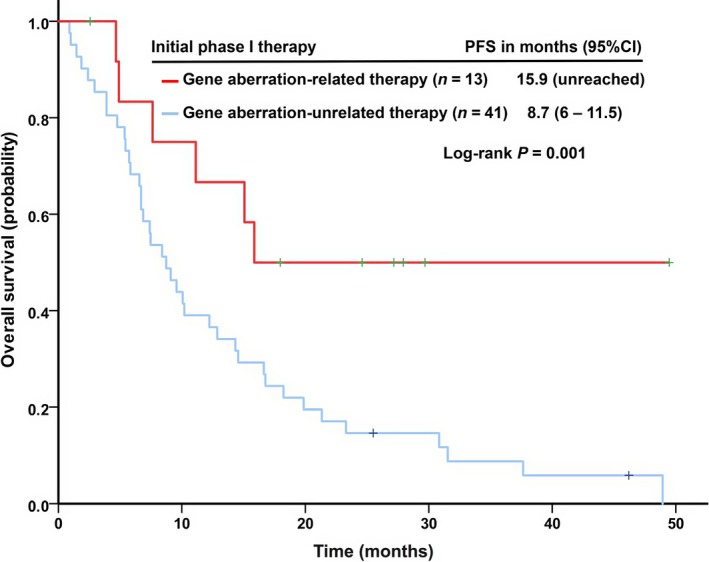
Kaplan–Meier curves of overall survival (OS) in 54 patients with metastatic leiomyosarcoma under the treatment of phase I clinical trials stratified by “gene aberration‐related” or “gene aberration‐unrelated” phase I therapies.

Patients who had received phase I therapy (*n* = 54) had a median OS of 10.4 months (95% CI: 6.9–13.9). Gene aberration‐related therapy during their initial phase I trial treatment (Fig. 2) yielded a significantly longer median OS (*n* = 13; 15.9 months [95% CI: unreached]) than gene aberration‐unrelated therapy (*n* = 41; 8.7 months [95% CI: 6–11.5]; *P *=* *0.013). A multivariable Cox proportional hazards model showed that hemoglobin <10 g/dL (*P *=* *0.015), body mass index (BMI) <30 kg/m^2^ (*P *=* *0.019), albumin <3.5 g/dL (*P *=* *0.001), and neutrophilia defined as neutrophils > upper limit of normal (*P *=* *0.014) were independently predictive of poor OS (Table 3).

**Table 3:** Univariate and multivariate analyses of OS after phase I therapy (*n* = 54)

Factors	Category	Univariate	*P*	Multivariate	*P*
		Median (months, 95% CI)		Hazard ratio (95% CI)	
Age ≤55 years	Yes (*n* = 28)	11.1 (5.38–16.9)	0.918	0.53 (0.22–1.28)	0.158
	No (*n* = 26)	9.1 (5.1–13.1)			
Caucasian	Yes (*n* = 36)	12.9 (7.7–18.1)	0.513	0.59 (0.26–1.37)	0.22
	No (*n* = 18)	6.9 (5.3–8.5)			
Site of origin (uterine)	Yes (*n* = 27)	10.1 (7.6–12.6)	0.565	0.38 (0.14–1.08)	0.068
	No (*n* = 27)	12.9 (3.6–22.1)			
ECOG performance status	0 (*n* = 12)	8.7 (6.2–11.2)	0.42	0.81 (0.29–2.27)	0.684
	≥1 (*n* = 42)	10.2 (5.4–15)			
Lactate dehydrogenase (≤618 IU/L)	Yes (*n* = 36)	14.6 (8–21.1)	0.07	2.16 (0.91–5.13)	0.081
	No (*n* = 18)	5.7 (3–8.5)			
Albumin (≥3.5 g/dL)	Yes (*n* = 51)	11.1 (6.8–15.5)	<0.001	43.59 (4.73–401.72)	0.001
	No (*n* = 3)	1.5 (0.6–2.4)			
Number of metastatic sites	≤ 2 (*n* = 28)	10.1 (8.2–11.9)	0.81	1.04 (0.44–2.41)	0.937
	>2 (*n* = 26)	8.7 (1.9–15.6)			
Liver metastasis	Yes (*n* = 26)	12.9 (2.6–23.2)	0.408	1.21 (0.52–2.82)	0.658
	No (*n* = 28)	9.6 (6.7–12.4)			
Hyperbilirubinemia	No (*n* = 54)	10.1 (6.2–14)	NA	NA	NA
	Yes (*n* = 0)	NA			
Creatinine (≤upper limit of normal)	Yes (*n* = 48)	10.2 (5.5–14.9)	0.122	2.68 (0.81–8.83)	0.105
	No (*n* = 6)	8.4 (0–17.8)			
Venous thromboembolism	Yes (*n* = 14)	5.4 (4.4–6.3)	0.11	2.43 (0.97–6.07)	0.058
	No (*n* = 40)	12.9 (7.5–11.2)			
Hemoglobin (≥10 g/dL)	Yes (*n* = 47)	12.2 (7–17.5)	0.015	5.62 (1.39–22.71)	0.015
	No (*n* = 7)	5.4 (3.5–7.4)			
Thrombocytopenia	No (*n* = 37)	9.6 (7.1–12)	0.999	0.61 (0.26–1.43)	0.255
	Yes (*n* = 17)	11.1 (4.9–17.3)			
Neutrophilia	No (*n* = 50)	10.1 (5.3–14.9)	0.705	0.09 (0.01–0.62)	0.014
	Yes (*n* = 4)	4.7 (0–10.8)			
Lymphocytosis	No (*n* = 36)	12.9 (5–20.7)	0.259	1.63 (0.71–3.76)	0.251
	Yes (*n* = 18)	7.4 (6.2–8.6)			
Body mass index (≥30 kg/m^2^)	Yes (*n* = 20)	16.6 (4.7–28.5)	0.038	3.33 (1.22–9.09)	0.019
	No (*n* = 34)	7.5 (4.3–10.7)			
Prior antiangiogenic therapy	Yes (*n* = 10)	15.9 (11.4–20.3)	0.548	0.5 (0.18–1.4)	0.186
	No (*n* = 44)	8.7 (5.8–11.7)			

ECOG, Eastern Cooperative Oncology Group.

**Figure 3:**
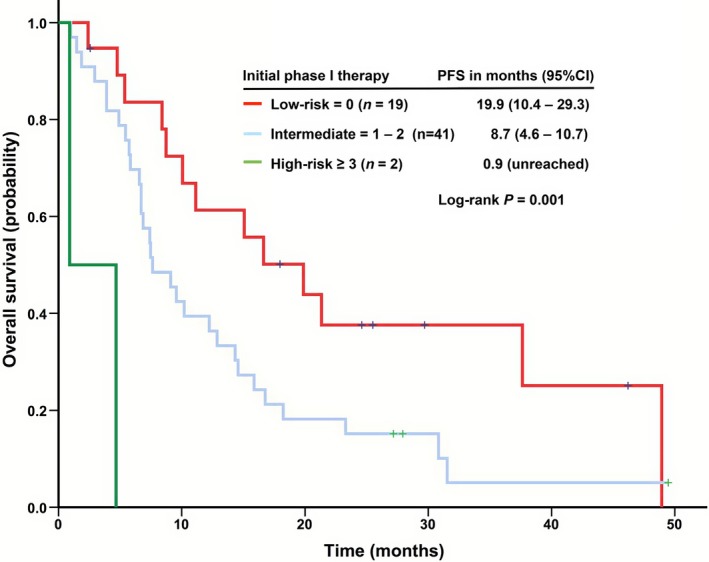
Kaplan–Meier curves of overall survival (OS) stratified by a prognostic scoring model (low‐risk [score = 0], intermediate‐risk [score = 1–2], and high‐risk [score ≥3] groups) in 54 patients with metastatic leiomyosarcoma who were treated in phase I clinical trials.

We determined whether the established prognostic scores were valid in this cohort of patients. The Royal Marsden Hospital Model was not able to categorize patients into distinct risk subgroups (*P *=* *0.224). When the MD Anderson Model was used, decreased survival was not associated with increased risk factors. Therefore, we further analyzed whether we can develop a new prognostic score model by using four independent risk factors (anemia, BMI <30 kg/m^2^, hypoalbuminemia, and neutrophilia) for predicting the survival of patients with metastatic LMS who were referred to a phase I service. Assuming the relative risks associated with each of the independent significant risk factors were comparable, the relative risk of death could be assessed by summing the number of risk factors present at the initial phase I clinic visit. Three risk groups were categorized (Fig. 3): low‐risk group (score=0; median OS, 19.9 months [95% CI: 10.4–29.3]), intermediate‐risk group (score=1 or 2; 7.6 months [95% CI: 4.6–10.7]), and high‐risk group (score ≥3; 0.9 months [95% CI: NA]; *P *<* *0.001).

Further survival analyses showed that patients who enrolled in their initial phase I trial before the end of 2010 (*n* = 24) had a median OS from their initial diagnoses of metastasis of 40 months (95% CI: 31.4–48.5) or from their initial phase I clinic visit of 6.9 months (95% CI: 4.7–9.1), significantly shorter than the median OS of those who did after 2010 (*n* = 30) who had a median OS from their initial diagnoses of metastasis of 65.4 months (95% CI: 36.1–94.7; *P *=* *0.008) or from the their initial phase I clinic visit of 15.1 months (95% CI: 6.8–23.4; *P *=* *0.036). Similarly, patients who died before the end of 2010 had a median OS from their initial diagnoses of metastasis of 40 months (95% CI: 32.9–47), which was significantly shorter than that of patients who died after 2010, who had a median OS from their initial diagnoses of metastasis of 52.4 months (95% CI: 25.5–79.2; *P *=* *0.01). When the cut‐off time was shifted to the end of 2012, the difference was significantly increased from a median OS of 39 months (95% CI: 31.8–46.2) in patients who died before the cut‐off time to 81.4 months (95% CI: 55.8–107; *P *<* *0.001).

## Discussion

To the best of our knowledge, this retrospective study is the largest cohort review of patients with metastatic LMS who participated in phase I clinical trials. Several lessons can be learned from this study.

### Potential benefit of using gene aberration‐related therapy for metastatic LMS

Development of LMS is characterized by the presence of complex and unbalanced karyotypes, resulting in genomic instability associated with multiple gene aberrations such as *TP53*,* RB1*,* CDKN2A*, IGFR, and *PTEN* 6, 29, 30, 31. These gene aberrations subsequently cause the activations of corresponding signaling pathways such as the RB1/cyclin D1, p53/MDM2, PI3K/AKT/mTOR, and IGFR/AKT pathways, which provided potential targets for future drug development 32, 33, 34, 35. The normal function of p53 protein inhibits angiogenesis during tumorigenesis, and enhanced angiogenesis occurs in tumors associated with *TP53* mutation through overexpression of vascular endothelial growth factor 36. Therefore, it might be appropriate to contemplate antiangiogenic therapy as gene aberration‐related therapy for the treatment of *TP53* mutant malignancies 25, 37, 38. We found that patients harboring the hotspot *TP53* mutation showed significantly better survivals with antiangiogenic‐based phase I trial therapy than did those without the hotspot *TP53* mutation. In this regard, patients who had received gene aberration‐related therapy achieved significantly greater antitumor activity, PFS, and OS than those who had not, suggesting that further investigation and classification of mutation profiling in LMS tumorigenesis may provide potential targets for drug development, which has started to change clinical practice for the treatment of metastatic LMS by using antiangiogenic‐based and/or gene aberration‐related therapeutic regimens (i.e., an mTOR inhibitor‐based therapy for a PIK3CA mutation or a PTEN aberration).

### Survival improves over time associated with availability of therapeutic options

Overall survival improvement in patients with metastatic colon cancer over time was found to be associated with increased use of new therapeutic agents 39. Thus, we determined whether survival duration in patients with metastatic LMS improved over time, similar to the findings in colon cancer since therapeutic agents have been made available for the treatment of metastatic LMS as well as improved best supportive care. Regardless of the cut‐off date we used (the end of 2010 or 2012), we found that overall survival duration improved in patients with metastatic LMS who ran out of therapeutic options and required phase I trial therapy, this improvement occurred over time, either from the date of their initial diagnosis of metastasis or from the date of their initial phase I office visit, was associated with increased availability of systemic therapeutic options. Preliminary evidence of the association between increased therapeutic options and improved survival in this cohort of patients with metastatic LMS suggests that it is imperative to make available to these patients all therapeutic agents that have established clinical benefits in metastatic LMS. Furthermore, when these patients run out of all standards of care options, they should be referred to novel phase I trial therapies to obtain maximum survival and clinical benefits. Although this does not appear to be what our data suggest, we will advocate that phase I trial referral earlier than all conventional options exhausted, especially when patients do not need urgent cytoreduction therapy, might be appropriate when gene aberration‐related or novel phase I trials are available.

### A new LMS prognostic scoring model predicts individual outcome

In phase I cancer patients, poor prognosis can be predicted by baseline risk factors, such as hypoalbuminemia, elevated LDH level, poor ECOG performance status, and the presence of more than two metastatic sites 40, 41. Accordingly, two prognostic scoring models were established for phase I cancer patients: the Royal Marsden Hospital prognostic scoring 40 and the MD Anderson prognostic scoring 41. However, in this cohort of phase I patients with metastatic LMS, we identified only one risk factor (hypoalbuminemia) in these models. Multivariate analysis of these patients revealed that four baseline parameters (anemia, BMI <30 kg/m^2^, hypoalbuminemia, and neutrophilia) predicted individual survival outcome. Therefore, it is appropriate to establish a new LMS prognostic score model to predict individual survival of phase I patients with metastatic LMS. With the use of the four identified risk factors mentioned above, we can subgroup phase I patients with metastatic LMS into low‐risk (score = 0), intermediate‐risk (score = 1 or 2), and high‐risk (score ≥3) groups. Since all these four risk factors are associated with the chronic inflammatory process, future drug development for the treatment of metastatic LMS might focus on therapeutic regimens modulating cross‐talk among inflammation, angiogenesis, immune response, and other biological pathways.

We recognize that the nature of this retrospective study raises concerns about bias in considering the clinical relevance and importance of our findings. Always inherent to retrospective methodology, the selection bias of patient referral to our phase I clinical trials program may limit the generalizability of our findings; thus, alternative theories may explain our findings. Small sample sizes limited the statistical validity and the actual conclusions could not be derived from our preliminary evidences. In this regard, conclusions from this retrospective study should be used for the purpose of hypothesis generation, which should be validated in larger prospective studies.

## Conclusions

All established standard‐of‐care therapeutic agents are made available to all LMS patients, however, efforts should be made to delineate biological and molecular characteristics of LMS during tumorigenesis and development to ensure that effective therapeutic agents continue to be developed. Potential therapeutic targets and prognosis should be identified in individual patients, and when these patients exhaust all standard‐of‐care options, referring them to a phase I trial therapy might provide survival and clinical benefits to some patients.

## Conflict of Interest

The authors report no conflicts of interest.
